# Extraction of Natural Gum from Cold-Pressed Chia Seed, Flaxseed, and Rocket Seed Oil By-Product and Application in Low Fat Vegan Mayonnaise

**DOI:** 10.3390/foods11030363

**Published:** 2022-01-27

**Authors:** Taha Hijazi, Salih Karasu, Zeynep Hazal Tekin-Çakmak, Fatih Bozkurt

**Affiliations:** 1 Department of Food Engineering, Faculty of Chemical and Metallurgical Engineering, Davutpasa Campus, Yildiz Technical University, Istanbul 34349, Turkey; hijazi8319@gmail.com (T.H.); zhazaltekin@gmail.com (Z.H.T.-Ç.); fbozkurt@yildiz.edu.tr (F.B.); 2 Department of Food Engineering, Engineering and Architecture Faculty, Muş Alparslan University, Muş 49250, Turkey

**Keywords:** cold-pressed oil by-product, gum, thixotropic behavior, low-fat vegan mayonnaise, thickeners, gelling agents

## Abstract

This study involves the modeling of rheological behavior of the gum solution obtained from cold-pressed chia seed (CSG), flaxseed (FSG), and rocket seed (RSG) oil by-products and the application of these gums in a low-fat vegan mayonnaise formulation as fat replacers and emulsifier. CSG, FSG, and RSG solutions showed shear-thinning flow behavior at all concentrations. The K values ranged between 0.209 and 49.028 Pa·s^n^ for CSG, FSG, and RSG solutions and significantly increased with increased gum concentration. The percentage recovery for the G′ was significantly affected by gum type and concentrations. CSG, FSG, and RSG showed a solid-like structure, and the storage modulus (G′) was higher than the loss modulus (G″) in all frequency ranges. The rheological characterization indicated that CSG, FSG, and RSG could be evaluated as thickeners and gelling agents in the food industry. In addition, the rheological properties, zeta potential, and particle size and oxidative stability (at 90 °C) of low-fat vegan mayonnaise samples prepared with CSG, FSG, and RSG were compared to samples prepared with guar gum (GG), Arabic gum (AG), and xanthan gum (XG). As a result, CSG, FSG, and RSG could be utilized for low-fat vegan mayonnaise as fat and egg replacers, stabilizers, and oxidative agents. The results of this study indicated that this study could offer a new perspective in adding value to flaxseed, chia seed, and rocket seed cold-press oil by-product.

## 1. Introduction

Edible oils contain ubiquitous dietary ingredients that may contain high quantities of bioactive compounds, such as sterols, tocopherols, and unsaturated fatty acids [1]. The cold-pressed extracted oils are not subjected to any refining and chemical processes. Also, cold-pressed oil by-products can be used in the food industry as they are not exposed to a solvent such as hexane. The moisture is removed before the processing of cold-pressed oil by-products and the oil is removed during production. The obtained byproducts are rich in proteins and polysaccharides. Today, although cold-press oil byproducts are an important source in terms of nutrition and technology, they have not taken their place in the food industry sufficiently. However, the development of new uses for cold-pressed oil byproducts of the edible oil industry by converting them to value-added products would prevent their disposal as waste and would encourage sustainable and competitive industrial supply [2]. Cold-pressed oil byproducts of seeds, such as flaxseed, chia seed, and rocket seed have an important potential in terms of natural gum. These byproducts have high gum content and can be easily extracted from food. 

Flaxseed (*Linum usitatissimum* L.) contains rich dietary fiber (~30% by weight), unsaturated fat (~40% by weight), and bioactive protein/peptides (~20% by weight) [3,4]. Cold-pressed flaxseed oil byproducts (FOB) contained 46.37% of carbohydrate, 27.67% of protein, 9.15% of oil, and 2.87% of ash on a wet basis [5]. Flaxseed gum (FG) is a natural polysaccharide and protein blend derived from flaxseed. FG is rich in soluble dietary fiber (3–9 wt% of the flaxseed) and can be used as a thickener, a stabilizer, a gelling agent, and an emulsifier [6,7,8]. 

Chia seed (*Salvia hispanica* L.) is rich in carbohydrates (~42%, mainly dietary fiber), proteins (17–24%), oil (25–40%), and omega-3/omega-6 fatty acids (60–80% of total oil) [9]. Chia seed polysaccharide (CSP), also known as chia seed gum (CSG), is a water-soluble anionic heteropolysaccharide isolated from the chia seed coat [10]. Chia seed contains about 5% mucilage, which can also act as soluble fiber [11]. Cold-pressed chia seed oil by-products obtained from the oil industry can be considered as a natural source of oil substitutes due to its high polysaccharide and protein content [12]. Chia seed gum can be used in a variety of industrial applications, including dietary fiber supplements, fat replacer, gelling agents, thickeners, stabilizers, emulsifiers, bulking agents, and film/coating agents [13]. 

Rocket seed (*Eruca sativa*) contains a variety of health-promoting phytochemical compounds, such as polyphenols, fibers, and glucosinolates. It has been used in the industry for oil production due to its high oil contents (20.0%) and Erucic acid used for the manufacture of a wide range of industrial products, e.g., plasticizers, surfactants, detergents, coatings, and polyesters. [14]. The rocket seed has a significant amount of total carbohydrate (23.1%), crude fibers (20.4%), and protein (31.0%) [15]. Thanks to their carbohydrate and protein content, rocket seeds have reasonable gum content with outstanding functional properties. Rocket seed gum (RSG) has high carbohydrate (80.38%) and low protein (5.81%) contents so the purification method used when obtaining gum could be appropriate. The protein content of gum is a crucial parameter determining its emulsion, foaming, and film-forming capacity. When comparing commercial gums, the protein content of RSG was higher than that of xanthan gum (2.125%), lower than that of guar gum (8.19%), and close to that of locust bean gum (5.2–7.4%) [16].

Mayonnaise is a semi-solid oil in water (O/W) emulsion that mainly consists of vegetable oil (70–80%), egg yolk, vinegar, and salt [17]. Egg yolk has an important place among the components to ensure the stability of mayonnaise and contributes to the overall organoleptic properties of the final product [18]. However, there has been a growing tendency toward substituting eggs with plant-based components, particularly in the creation of mayonnaise analogs, due to health and environmental concerns, recently. Egg yolk is an important ingredient that affects oil droplet distribution and emulsion stability in emulsion products, such as mayonnaise. Therefore, it is very difficult to find an alternative stabilizing agent to egg yolk in vegan mayonnaise production. In recent years, the number of studies on alternatives to egg yolk has been increasing. In this study, low-fat vegan mayonnaise samples were prepared using CSG, FSG, RSG, AG, GG, and XG, and rheological properties were compared. 

There are many studies on the rheological properties of different gum solutions. However, the researchers focused more on the steady and dynamic rheological properties of the gums. The number of studies on the thixotropic properties of natural gums is limited. In this sense, there are important gaps in the literature. In this study, natural gums were produced from the cold-pressed chia seed, flaxseed, and rocket seed oil by-products. This study will also demonstrate the thixotropic behavior of a wide range of gums using specific rheological tests such as 3-ITT. Also, gums produced from cold-pressed oil by-products can be used as an alternative emulsifier for oil in water (O/W) emulsions such as mayonnaise and salad dressings to obtain a more stable O/W emulsion having expected quality. In this study, by-product gums were utilized as egg yolk replacers to produce vegan mayonnaise.

## 2. Materials and Methods

In this study, cold-pressed oil byproducts obtained from chia seed (*Salvia hispanica* L.), flaxseed (*Linum usitatissimum* L.), and rocket seed (*Eruca sativa*) were supplied from ONEVA Food Co. (Istanbul, Turkey) for gum extraction. Ethanol and all other chemicals and standards to be used during gum extraction are of analytical quality and purchased from Merck (Darmstadt, Germany) or Sigma-Aldrich (St. Louis, MO, USA). Sunflower oil and sunflower lecithin, distilled water, CSG, FSG, RSG, guar gum (GG), Arabic gum (AG), and xanthan gum (XG) were used for low-fat vegan mayonnaise preparation. 

### 2.1. Gum Extraction

Gums were extracted from cold-pressed oil by-products (chia seed, flaxseed, and rocket seed) according to the procedure described by Naji-Tabasi et al. [19]. The gums were removed from the by-products by soaking with water followed by ethanol precipitation. Five hundred by-products are weighed and added to 10 L of distilled water and mixed with a magnetic heated stirrer at 80 °C for 2 h to obtain gel structures that come out of the byproducts. The extracted gum solution from the byproducts was passed through to the sieve and then purified by mixing with three volumes of 96% ethanol to precipitate gum and left in the oven at 30 °C for 1 day to dry. The seed gums are dried, packaged at 4 °C, and stored in dry conditions. 

### 2.2. Gum Characterization

The moisture contents of CSG, FSG, and RSG were determined by drying the sample at 105 °C for 5 h in a conventional oven (Memmert UF-110, Schwabach, Germany) [20]. The ash contents were determined by burning of the organic material of the sample at 550 °C in a muffle furnace (WiseTherm-Daihan FH-03, Seoul, Korea) for 6 h [21]. The protein contents were determined by the Kjeldahl method using the Behr Kjeldahl unit (Unit-S5, Ahlen, Germany) with the conversion factor of 6.25 [22]. The fat contents were analyzed by Soxhlet extraction using hexane as a solvent [23]. 

The monosaccharide (glucose, galactose, mannose, and xylose) compositions of CSG, FSG, and RSG were determined using an HPLC tool (Agilent 1260 Infinity) equipped with Rezex ROA-Organic Acid H+ (New Column, 300 × 7.8 mm). A 250-mg gum sample was heated with 2 M of H_2_SO_4_ for 2 h at 120 °C. Then, the hydrolyzed mixture was cooled, the pH of the solution was adjusted to 7 using 5 M of NaOH and the final volume was adjusted to 10 mL with distilled water. Then, it was filtered through a 0.45-μm syringe filter and injected into the column [24].

ATR-FTIR (attenuated total reflection-Fourier transform infrared) spectroscopy (Bruker Tensor 27, Borken, Germany) equipped with a KBr beam diffuser and DLaTGS detector was used to characterize CSG, FSG, and RSG. ATR-FTIR spectra of CSG, FSG, and RSG with wavenumbers ranging from 400 to 4000 cm^−1^ were acquired with 16 scans per spectrum and 2 cm^−1^ resolutions. Deconvolution was applied to all spectra by interpretation of changes in the overlapped amide I band (1600–1700 cm^−1^) using Origin 2020b software to determine changes in the secondary structure of hydrolysates [25].

### 2.3. Preparation of Gum Solutions

Gum solutions were prepared at concentrations of 1.0–2.0% using flaxseed and chia seed gums, and 1.0–5.0% for rocket seed gum. First of all, natural gums dissolve in water at a certain concentration and were expected to mix in a magnetic stirrer for 12 h to fully hydrate. Each measurement was repeated three times.

### 2.4. Rheological Properties

The flow behavior and thixotropic and dynamic rheological properties of gum solutions were determined using a temperature-controlled rheometer (MCR 302, Anton Paar, Graz, Austria). All rheological measurements were carried out at 25 °C.

### 2.5. Flow Behavior Rheological Properties

The flow behavior characteristics of the gum solutions were determined in the range of 0–100 shear rate (1/s) using a parallel plate configuration and a 0.5-mm gap between the rheometer probe and the sample plate. Apparent viscosity values corresponding to shear stress and shear rate were recorded. The flow behavior rheological properties were modeled using the Herchel–Bulkley model by nonlinear regression.

(1) $$\tau =K{\left(\gamma \right)}^{n}$$

In Equation (1), *τ* is the shear stress (Pa), *K* is the consistency coefficient (Pa·s^n^), *γ* is the shear rate (s^−1^), and *n* is the flow behavior index (dimensionless). 

### 2.6. Thixotropic Properties

#### 2.6.1. Constant Shear Rate

Shear stress or *η*50 values were used to determine the time-dependent behavior of gum solutions at 25 °C. The experimental data could be fitted to the Weltman model (Equation (2)) and second-order structural kinetic model (Equation (3)), with *n* = 2 [26,27].

(2) σ=a+blnt

In Equation (2), *σ* is the shear stress at time *t* (Pa), *a* is the initial shear stress (Pa), *b* is the time coefficient of thixotropic breakdown (Pa), and *t* is the time of shearing (s).

(3) $${\left[\frac{\eta -\eta_{e}}{\eta_{0}-\eta_{e}}\right]}^{\left(1-n\right)}=(n-1)kt+1$$

In Equation (3), *η*_0_ is the initial apparent viscosity at *t* = 0 (structured state), *η_e_* is the equilibrium apparent viscosity as t→∞ (non-structured state), *k* = *k*(*γ*) is the rate constant, and *n* is the order of the structure breakdown reaction assumed as 2 in the second-order structural kinetic model. 

#### 2.6.2. 3-ITT Rheological Properties

The 3-ITT rheological properties were determined in the range of 0.5/s constant shear rate and 150/s variable shear rate, respectively. When the values were selected, the linear viscoelastic region was taken into account, and the linear viscoelastic region of the samples ends at 50 s^−1^. Gum solutions were subjected to a very low shear rate (0.5/s) for 100 s during the first-time interval. In the second time interval, it was subjected to the specified shear force for 40 s. In the third time interval, the dynamic rheological behavior in the second time interval was determined by exposing the samples to the low shear rate in the first time interval. The second-order structural kinetic model was used, and *G*_0_, *G_e_*, and *k* values were calculated by the following Equation (4):

(4) $${\left[\frac{G^{\prime }-G_{e}}{G_{0}-G_{e}}\right]}^{1-n}=\left(n-1\right)kt+1$$

In Equation (4), *G*_0_ (the initial values of the storage modulus in the first interval), *G_e_* (the equilibrium storage modulus as *t* → ∞), *k* (the rate constant of recovery of the sample) and in this model *n* = 2, are specified. 

In Equations (5) and (6), *G_i_* (at the initial state of the product), *G*_0_ (after deformation applied *G*′ value), and *G_e_* (after recovery of sample *G*′ value) values are characterized by deformation equation [28]:

(5) $$\%\;Deformation=\left(\;\frac{\left(G_{i}-G_{0}\right)}{G_{i}}\;\right)\times 100$$

The recovery degrees of CSG, FSG, and RSG is determined by Equation (6),

(6) $$\%\;Recovery=\left(\frac{G_{e}}{G_{i}}\right)\times 100$$

#### 2.6.3. Dynamical Rheological Properties

The dynamic rheological properties of the gum solutions were carried out using a parallel plate configuration. First, the amplitude sweep test was performed with a strain value of 0.1% to determine the direct viscoelastic region. The frequency sweep test was applied in the range of 0.1–10 Hz and 0.1–64 (*ω*) angular velocity in the primary viscoelastic region. The values of storage modulus (*G*′) and loss modulus (*G*″) were measured against angular velocity and frequency. The parameters related to dynamic rheological properties were determined using the Oswald-de Waele model and nonlinear regression [29].

(7) $$G^{\prime }=K^{\prime }{\left(\omega \right)}^{n^{\prime }}$$

(8) $$G^{\prime \prime }=K^{\prime \prime }{\left(\omega \right)}^{n^{\prime }}$$

Equations (7) and (8), *G*′ value corresponds to storage modulus (Pa), *G*″ value to lose modulus (Pa), *ω* value to angular velocity value (s^−1^), *n*′ and *n*″ values to flow behavior index values, and *K*′ and *K*″ values to consistency coefficient (Pa·s^n^).

### 2.7. Vegan Mayonnaise Preparation and Analysis

The different types of gum (CSG (2%), FSG (2%), RSG (5%), Arabic gum (AG:1%), guar gum (GG:1%), and xanthan gum (XG:0.4%) were used in vegan mayonnaise production. Firstly, the gums were dispersed at 25 °C in water at different ratios. Afterward, the gum was hydrated by stirring at 1000 rpm in a magnetic stirrer for 6 h. The obtained dispersion was combined with sunflower oil (30%) and lecithin (1%) and homogenized for 3 min utilizing Ultra Turrax (Daihan, HG15D) at 10,000 rpm. Finally, low-fat vegan mayonnaise samples were obtained. 

#### 2.7.1. Rheological Analysis

All rheological analyses, i.e., flow behavior properties, dynamic rheological proper-ties, and 3-ITT rheological properties of vegan mayonnaise prepared with byproduct gums, were determined using a temperature-controlled rheometer (MCR 302, Anton Paar, Austria) at 25 °C.

#### 2.7.2. Zeta Potential and Particle Size

The zeta(ζ-)-potential, the oil particle size (d_32_), and polydispersity index (PdI) were measured by a particle size meter (Nanosizer, Malvern Instruments, Malvern, Worcestershire, UK) with electrophoresis and dynamic light scattering system in the continuous phase of the mayonnaise samples. Until the measurement, the samples were diluted 500 times with ultrapure water and then homogenized by mixing in an ultrasonic water bath. This procedure was repeated in triplicate for each sample by using the Zeta potential measurement, and the averages of the values and the standard deviations were determined. 

#### 2.7.3. Oxidative Stability (OXITEST)

The oxidative stability of the mayonnaise samples was tested using the OXITEST Device (Velp Scientifica, Usmate, Italy) according to AKSOY et al. [30]. All samples were weighed before the oxidative stability analysis started. Firstly, 20 g of each mayonnaise sample were weighed into the sample cells. The device temperature and the oxygen pressure were adjusted at 90 °C and 6 bars, respectively. The induction period (IP) value obtained by the OXITEST system was used to evaluate the oxidative stability values of the samples.

### 2.8. Statistical Analysis

The statistical analysis was carried out using the Statistica software program (StatSoft, Inc., Tulsa, OK, USA). All the rheological analyses were conducted in triplicate. The standard deviation and mean values were presented. ANOVA was conducted to determine the differences in rheological parameters of gum solutions. Duncan, multiple comparison tests at 95% significance level were used to determine the effect of analysis parameters. 

## 3. Results and Discussion

### 3.1. Characterization of Gum

Table 1 showed physicochemical properties of CSG, FSG, and RSG. CSG consisted of 73.59% carbohydrate, 9.45% protein, 1.01% fat, 9.60% moisture, 6.26% ash (%*w*/*w*). FSG contained 78.56% carbohydrate, 11.38% protein, 2.16% fat, 9.08% moisture, 7.89% ash (%*w*/*w*) while RSG consist of 60.48% carbohydrate, 21.00% protein, 1.94% fat, 9.95% moisture, 6.63% ash (%*w*/*w*). 

The protein content of gums is a very important parameter affecting the emulsification and foaming ability of gums. The protein content of the obtained gums was found to be higher than other natural and commercial gums [16,31]. RSG is rich in galactose and mannose, while FSG and CSG are rich in xylose and glucose. It can be said that RSG has a galactomannan structure as in many gums. The ratio of mannose to galactose was found to be 1.77 in RSG. The mannose to galactose ratio strongly affected the technological properties of gums such as their cold-water solubility, thickening, gelling, and crystalizing properties. The mannose galactose ratio was lower than guar gum (2:1) and LBG (4:1), and higher than fenugreek gum (1:1) [32,33]. In a similar study, xylose and glucose were reported to be higher than mannose and galactose from studies on the sugar profile of mucilage and gums obtained from flaxseed and chia seeds [34,35,36,37]. 

**Figure 1:**
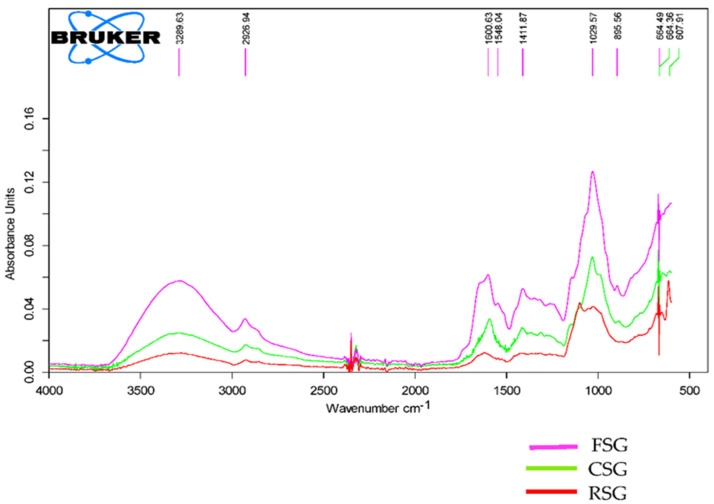
FTIR spectra of the FSG (Flaxseed byproduct gum), CSG (Chia seed byproduct gum), and RSG (Rocket seed byproduct gum) in the spectral region between 400 and 4000 cm^−1^.

The FTIR spectrum of CSG, FSG, and RSG is illustrated in Figure 1. The results revealed that all gum samples showed a similar FTIR spectrum with some minor differences. This difference might be due to their protein content and different sugar and organic acid composition. Characteristic bands varying between 3500–3100 cm^−1^ are attributed to the hydroxyl (-OH) stretch that forms the gross structure of carbohydrates [38]. The bands between 3000–2800 cm^−1^ represent -C-H stretching of the aromatic rings and the methyl group (CH3) [38]. The bands at 1654 cm^−1^ and 1618 cm^−1^ for chia seed and its mucilage are assigned to a mannose ring [39].The bands at around 1597 cm^−1^ and 1422 cm^−1^ are due to the carboxyl groups of uronic acid residues in the gum polysaccharide or the presence of protein in the gum samples [40]. This result is compatible with the protein content of the gums. The wavenumber between 950 cm^−1^ and 1200 cm^−1^ is generally considered the fingerprint region of polysaccharides where the main chemical groups in polysaccharides are identified. The band at around 1030 cm^−1^ is assigned to C-O-C stretching of 1→4 glycosidic bonds [41]. Also, the strong absorption at 1014 cm^−1^ shows the stretching vibration of the C–N [42]. The band at 864 cm^−1^ is assigned to the β-anomeric C-H deformation and glycosidic linkages of glucopyranose and xylopyranose units [38]. The FTIR spectrum obtained is very similar to many mucilage and gums studied in the literature. Hadad and Goli [43] observed the FTIR spectrum of flaxseed mucilage gave the absorption peaks at 3321, 2922, 1612, 1410, and 1050 cm^−1^. Darwish and El-Sohaimy [44] reported the absorption peaks of chia seed mucilage at 1739, 1539, 1444, 1419, 1157, 1058, and 618 cm^−1^. 

### 3.2. Flow Behavior Rheological Properties of the CSG, FSG, and RSG Solutions

**Figure 2:**
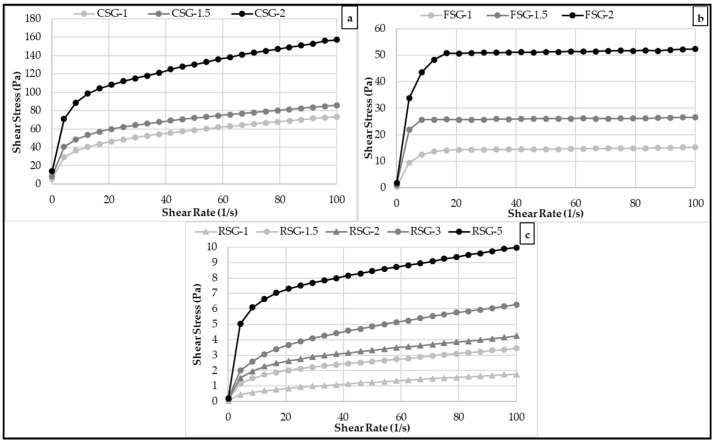
Steady shear rheological properties of the FSG (flaxseed byproduct gum), CSG (chia seed byproduct gum), and RSG (rocket seed byproduct gum) solutions with different concentrations ((**a**) CSG (1–2%), (**b**) FSG (1–2%), (**c**) RSG (1–5%)).

Figure 2 exhibits the flow properties of CSG (1.0, 1.5, and 2.0 %, *w*/*w*), FSG (1.0, 1.5, and 2.0 %, *w*/*w*), and RSG (1.0, 1.5, 2.0, 3.0, and 5.0 %, *w*/*w*) solutions with different concentrations over the range of shear rate from 0.01 to 100 s^−1^ at 25 °C. The decrease in viscosity by an increase in shear rate indicated a non-Newtonian shear-thinning (pseudoplastic) flow behavior for CSG, FSG, and RSG solutions at all concentrations. The decrease in the viscosity values of the samples due to the increasing shear rate can be explained by the breaking of the weak bonds between the molecules in the product as a result of the applied force and the weakening of the interaction between the components [45,46]. As predicted, the constant shear viscosity increased with the increase of gum concentrations. The most used hydrocolloids in the food industry, such as locust bean gum [47] and xanthan gum [48], showed shear-thinning rheological behavior. Similar shear-thinning flow behavior of gum dispersions was previously reported by Sanchez et al. [49], Marcotte et al. [50], Yamazaki et al. [51], Razavi et al. [52], and Chaharlang and Samavati [53]. CSG and FSG dispersions showed high viscosity and pronounced shear-thinning behavior at low concentrations (1–2%); however, more RSG exhibited weak pseudo-plasticity at lower concentrations. This might be explained by the varied origins of these gums. The gums with high molecular weight and strong intermolecular interactions have high viscosity solutions [54]. The orientation effect is another explanation for the shear thinning behavior. As the shear rate increases, the randomly distributed polymer molecules become more and more aligned in the flow direction, resulting in reduced contact between neighboring polymer chains [55]. 

Shear-thinning gums are commonly used to enhance the texture and rheological properties of food products. The high-shear processing operations, such as pumping and filling cause a reduction in the apparent viscosity of the solution. However, the high apparent viscosity produces a pleasant tongue feel during consumption [56]. 

The power-law model parameters (the consistency index (K) and the flow behavior index (n)) are shown in Table 2. The power-law model was successfully applied for the modeling of flow behavior properties of CSG, FSG, and RSG solutions (The coefficients of determination: R^2^ > 0.96). Many previous investigations have shown that the power-law model was the suitable model for describing the flow behavior of gum solutions [47,50,56,57]. 

The K values were found as 0.209–49.028 Pa·s^n^. The increase of gum concentrations increased K values for all types of gum solutions. At the same concentration, CSG had the highest K values, followed by FSG and RSG. The n values of CSG, FSG, and RSG solutions at all concentrations were less than 1, indicating that all gums showed a non-Newtonian shear-thinning behavior (Table 2). All the gum solutions had strong shear-thinning behavior with *n* values as low as 0.143–0.460, indicating a significant deviation from Newtonian behavior, and they, like many other shear-thinning hydrocolloids, have a high viscosity and pleasant mouthfeel at low shear rates. CSG, FSG, and RSG samples exhibited shear-thinning behavior like locust bean gum [47], monoi gum [57], guar gum [58], and xanthan gum [48], which was explained to be due to weak bonds formed as a result of shearing [59]. When the n number is smaller than 0.6, it has been observed that non-Newtonian behavior becomes relevant [60]. The increase of gum concentrations caused decreasing n values. These results demonstrated that different concentrations of CSG, FSG, and RSG solutions affected the steady shear properties of CSG, FSG, and RSG solutions. 

**Figure 3:**
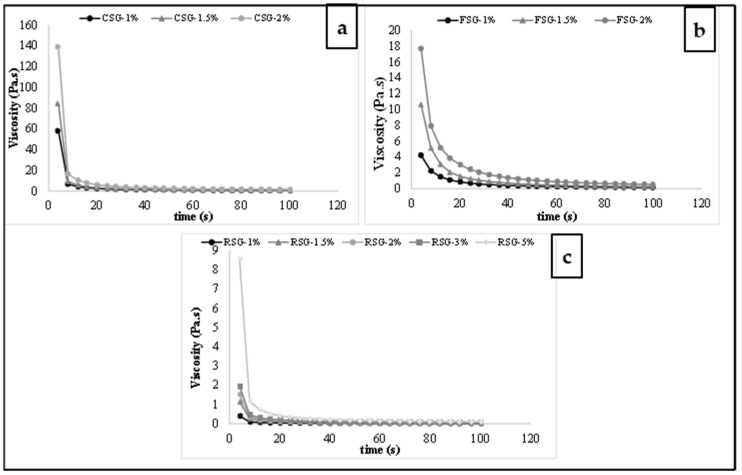
The viscosity of CSG: chia seed byproduct gum, FSG: flaxseed byproduct gum, RSG: rocket seed byproduct gum solutions with different concentrations ((**a**) CSG (1–2%), (**b**) FSG (1–2%), (**c**) RSG (1–5%)).

Figure 3 shows the viscosity of CSG, FSG, and RSG solutions with different concentrations over the range of shear rate from 0.01 to 100 s^−1^. As seen in Figure 2, the viscosity of the CSG, FSG, and RSG samples decreased with an increase in the shear rate at all concentrations due to the pseudoplastic behavior of gum solutions. The increase in shear rate led to a breakdown of molecular bonds, and therefore molecules became regular and internal friction decreased. As a result, the viscosity of CSG, FSG, and RSG solutions decreased. As the gum became entangled in the solution, the viscosity of the gum solutions increased with gum concentration, as can be seen in Figure 3. The higher viscosity value in CSG-2 (a solution containing 2% chia gum) compared to other gum solutions was associated with the strong interactions in hydrogen bonds [61].

### 3.3. Thixotropic Properties

#### 3.3.1. Constant Shear Rate

**Figure 4:**
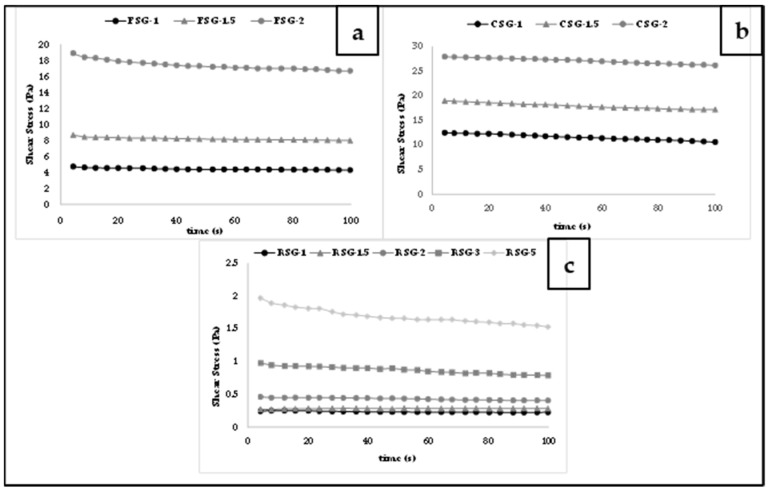
Shear stress vs. time at a constant shear rate (0.5 s^−1^) for CSG: chia seed byproduct gum, FSG: flaxseed byproduct gum, RSG: rocket seed byproduct gum solutions with different concentrations ((**a**) CSG (1–2%), (**b**) FSG (1–2%), (**c**) RSG (1–5%)).

CSG, FSG, and RSG solutions were sheared at a constant shear rate of 0.5 s^−1^ at 25 °C in the shear decay test. At 25 °C, Figure 4 depicts the change in shear stress of CSG, FSG, and RSG solutions as a function of time. Following that, the data were fitted to two different models: the Weltmann [62] model and the second-order structural model [63]. 

The computed parameters and their related determination coefficients are shown in Table 3. Both models could accurately reflect the time-dependency of shear stress values at all gum concentration levels with good R^2^ values. 

The second-order structural model provides information on the change in time-dependent flow characteristics caused by shearing, as well as the rate of breakdown, based on the sample’s structured and nonstructured state. The rate constant (k) represents the rate of structural breakdown (thixotropy degree), whereas the initial-to-equilibrium viscosity ratio (*η*_0_/*η_e_*) offers a relative assessment of structural breakdown quantity [26]. The parameters of the second-order structural model are shown in Table 3. Gum type also affected the magnitudes of k values and *η*_0_/*η_e_* values. As can be expected, *η*_0_ and *η_e_* values of gum solutions were increased with the increase of gum concentrations. Moreover, the k values and *η*_0_/*η_e_* ratios of gum solutions were increased with the increase of gum concentrations. The highest k and *η*_0_/*η_e_* values belong to CSG solutions, indicating that CSG solutions showed a faster rate of thixotropic breakdown and the extent of thixotropy. 

The Weltman model predicted well the relationship between the shear stress and shearing time of gum solutions, and its parameters (A and B) were utilized to examine the effect of temperature on stress decay behavior (Table 3). Parameter A represents the shear stress threshold for structural breakdown, while the time coefficient-B represents the amount of structural breakdown caused by applied shear [64]. Negative B values indicate how soon the apparent viscosity achieves equilibrium [60]. Lower B values imply that the structure of a product is less affected by stirring. Therefore, RSG and FSG were affected less than CSG by stirring due to their lower B values. The Weltman model’s coefficients were affected by the gum concentrations and applied constant shear rate [65]. 

#### 3.3.2. Three Interval Thixotropic Time Test (3-ITT) 

**Figure 5:**
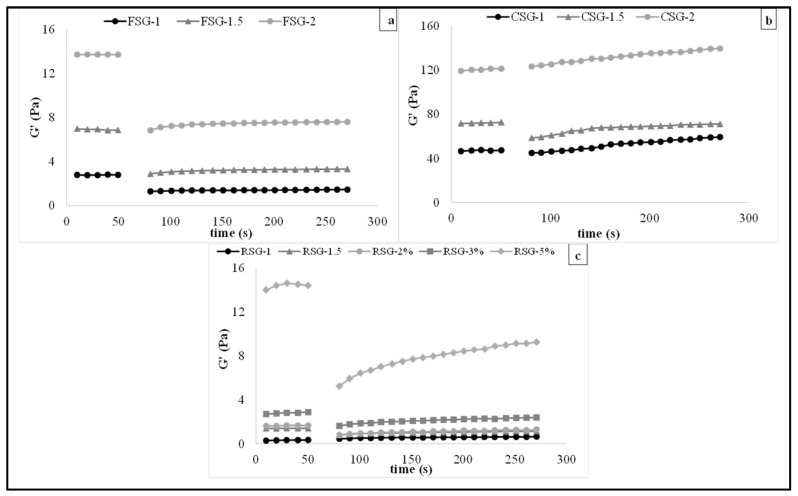
3-ITT rheological properties of CSG: chia seed byproduct gum, FSG: flaxseed byproduct gum, RSG: rocket seed byproduct gum solutions with different concentrations ((**a**) CSG (1–2%), (**b**) FSG (1–2%), (**c**) RSG (1–5%)).

Figure 5 showed the structural recovery of CSG, FSG, and RSG solutions by 3-ITT, which simulates the sudden and nonlinear deformation of gum solutions. The structural recovery tendency of CSG, FSG, and RSG solutions increased with the increase of gum concentrations. The lowest concentrations of gum solutions caused the lowest structural recovery. These results showed that the structural breakdown observed in the second time interval due to high sudden shear force could be easily recovered as the gum concentration increased [66].

Table 4 indicates the structural deformation and recovery ratio determined by fitting 3-ITT rheological data using a second-order structural model. The thixotropic constant (k), initial storage modulus (*G*_0_), equilibrium storage modulus (*G_e_*), and the ratio of *G_e_* and *G*_0_ (*G_e_*/*G*_0_) were calculated by the second-order structural kinetic model. The *G_e_*/*G*_0_ value represents the recovery percentage; the larger it is numerically, the faster it can be evaluated to tend to recover. *G_e_*/*G*_0_ values were between 1.124 and 1.750. CSG had the highest *G_e_*, *G*_0_, and *G_e_*/*G*_0_ values, indicating that CSG solutions were the higher recoverable character. As seen in Table 4, the k value indicates the thixotropic rate of samples, and a higher k indicates a higher recovery rate. For each type of gum, the higher gum content showed a higher k value. Samples containing the highest gum concentration showed the highest k and *G_e_*/*G*_0_, indicating that the sample showed the highest thixotropic behavior and viscoelastic solid character. Also, FSG-2 had the highest k value.

### 3.4. Viscoelastic Behavior of the CSG, FSG, and RSG Solutions

**Figure 6:**
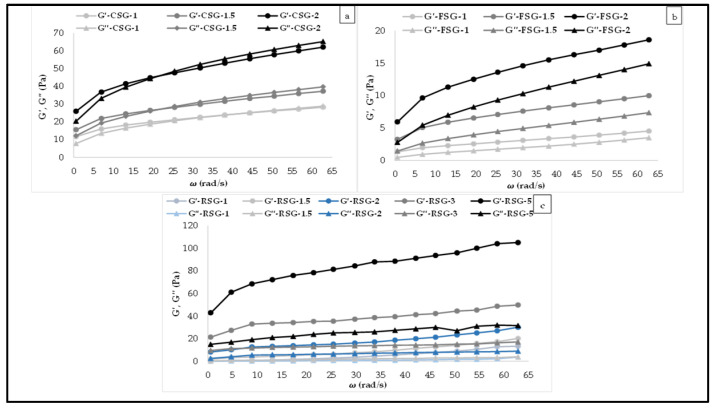
Viscoelastic behavior of the (**a**): CSG: chia seed byproduct gum (1–2%), (**b**): FSG: flaxseed byproduct gum (1–2%), (**c**): RSG: rocket seed byproduct gum (1–5%) solutions with different concentrations.

A frequency sweep test was utilized for the determining viscoelastic behavior of gum solutions. Figure 6 indicated the dynamic viscoelastic characteristics of CSG, FSG, and RSG solutions with different concentrations. As seen, the changes in storage (G′) and loss (G″) values for gum solutions were demonstrated as a function of angular frequency (*ω*) at 25 °C. The structure of the gum solutions may be shown in dynamic mechanical spectra, which reveal the frequency dependence of storage modulus G′ and loss modulus G″. There were no cross points between G′ and G″ values, revealing that G′ values were greater than G″ across the entire frequency range studied. As a result, gum solutions had typical gel-like behavior within the experimental frequency range at 25 °C. This viscoelastic behavior was in good agreement with that reported by Chaisawang and Suphantharika [67] and KUTLU et al. [68]. 

Non-linear regression was used to analyze experimental G′ and G″ values vs. *ω*, and the computed magnitudes of slopes (n′ and n″), intercept (K′ and K″), and coefficient of determination (R^2^) values are shown in Table 5. K″ values were determined to be higher than K′ values, indicating that gum solutions exhibited liquid-like characteristics. Similar results were reported in previous studies on different gum solutions [66,67,68] and different food products [69,70]. All of the solutions exhibited gel-like behavior due to the positive slopes (n′ and n″ > 0). The n′ value may be used to determine the strength and type of the gel; n′ = 0 indicates a covalent gel, while n′ > 0 indicates a physical gel. Low n′ values (around zero) indicate that G′ does not vary with frequency, but n′ values close to 1 indicate that the system acts like a viscous gel.

### 3.5. Rheological Properties, Zeta Potential, and Particle Size and Oxidative Stability of Low-Fat Vegan Mayonnaise Samples with Different Types of Gum

#### 3.5.1. Rheological Properties

**Figure 7:**
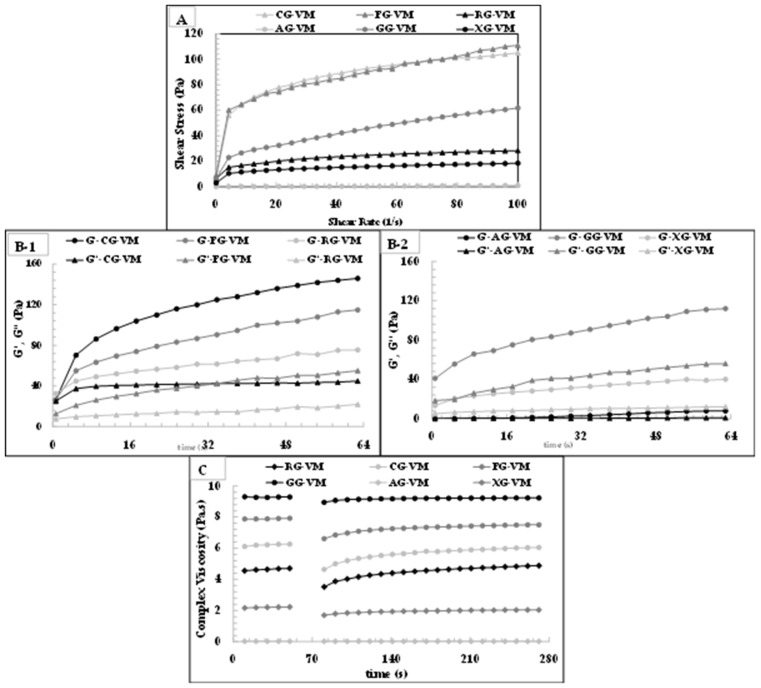
Rheological properties of vegan mayonnaise samples formulated with different gums. FG-VM: flaxseed oil byproduct gum vegan mayonnaise, CG-VM: chia seed oil byproduct gum vegan mayonnaise, RG-VM: rocket seed oil by-product gum vegan mayonnaise, GG-VM: guar gum vegan mayonnaise, XG-VM: xanthan gum vegan mayonnaise, AG-VM: gum Arabic vegan mayonnaise. (**A**) Steady shear rheological properties of the low-fat vegan mayonnaise samples, (**B-1**) Viscoelastic behavior of the low-fat vegan mayonnaise samples formulated with CG, FG, and RG, (**B-2**) Viscoelastic behavior of the low-fat vegan mayonnaise samples formulated with AG, GG, and XG, (**C**) 3-ITT rheological properties of the low-fat vegan mayonnaise samples.

According to the rheological characterization results, RSG, FSG, and CSG gave the highest K, K′, recovery values and thixotropic degree at the highest concentrations used. RSG, FSG, and CSG were compared with commercial gums at these concentrations. In determining the concentrations of commercial gums in mayonnaise production, the concentrations that gave successful results in the literature were taken into account. The steady shear rheogram of vegan mayonnaise samples was shown in Figure 7, which was absolute evidence of shear-thinning behavior (pseudoplastic behavior) of vegan mayonnaise. This typical behavior was reported for vegan mayonnaise by other researchers [71,72]. It can be concluded from Figure 7 that the mayonnaise sample that formulated the CG and FG had the highest shear-thinning behavior, followed by GG. 

The viscoelastic characteristics of the vegan mayonnaise samples obtained by frequency sweep measurement were characterized and shown in Figure 7. As seen, G′ was greater than G″ across the measured frequency range and both G′ and G″ were hardly influenced by frequency change (Figure 6). It can be concluded that all vegan mayonnaise samples showed more solid-like properties. This conclusion was conducted by [73] for mayonnaise formulated with micronized konjac gel. CG-VM samples had the highest G′ and G″ values. 

Figure 7 indicated the 3-ITT rheological properties of the vegan mayonnaise samples. Due to deformations during high-speed mixing and homogenization, as well as during consumption, such as when the packed food is shaken or pressed, thixotropic behavior is crucial for O/W emulsions, notably in vegan mayonnaise with low oil content. As can be observed in Figure 7, all samples showed thixotropic behavior in the third interval. Mayonnaise samples lost their viscoelastic characteristics after severe shear deformation but recovered them after a second period. These findings suggested that all mayonnaise samples may maintain their viscoelastic character throughout food processing involving a large amount of abrupt deformation, such as homogenization or pumping, as well as consumption under shaking and squeezing. This is the ideal flow behavior for mayonnaise. 

Table 6 presented the rheological properties of low-fat vegan mayonnaise samples prepared with a different type of gum. The flow parameters of the flow index (n), consistency index (K), and coefficient of determination (R^2^) were shown in Table 6. The n values were 0.208–0.769, indicating that all mayonnaise samples expected for AG-VM were pseudoplastic fluids (*n* < 1). The n values were reported as lower than 1 for some commercial mayonnaises and vegan mayonnaise samples [74,75,76,77]. K values of vegan mayonnaise samples were determined between 0.007 and 38.582 Pa·s^n^. CG-VM and FG-VM samples had similar and highest K values, explaining the strongest non-Newtonian behavior. 

Table 6 indicated the dynamic power-law parameters of vegan mayonnaise samples. K′ and K″ values of samples were found as 0.012–43.317 Pa·s^n^ and 0.006–30.423 Pa·s^n^, respectively. For the generation of dense viscoelastic interfacial networks at the air/water interface, neutral protein-polysaccharide complexes are recommended. These networks can minimize a thin-gas film’s permeability while also promoting foam stability, resulting in much lower interfacial area loss and air bubble coalescence rates. While unevenly charged protein-polysaccharide solutions can stabilize electrostatic repulsion forces between droplet surfaces and induce stability against flocculation and creaming of emulsions, they can also cause flocculation and creaming. 

#### 3.5.2. Zeta Potential and Particle Size

Zeta (ζ) potential is an important parameter that shows whether O/W emulsions can remain stable for a long time. As the ζ-potential value moves away from 0, in other words, the system having a negative or positive charge is a positive indicator for the long stability of the product. Table 7 showed that the ζ-potential values of the samples were found between (−42.80) and (−31.90) mV. The first interpretation we can make by looking at these values is that the ζ-potential value of all samples is higher than 0 or that the samples are stable products to a certain degree. The absolute ζ-potential values of our vegan mayonnaise samples were similar except for AG-VM, indicating that the samples can remain stable in these formulations for a long time. The fact that gum forms a compact structure by reducing the mobility of the mobile phase in increasing the stability of mayonnaise samples, thus reducing the action potential of the oil molecules in this tight structure and restricting the interaction of the droplets play a primary role [77]. The fact that the oil droplets have a certain electrical potential and interact with each other thanks to the electrostatic repulsive force, preventing flocculation. The zeta potential of samples prepared with gums obtained from byproducts is similar to commercial gums, indicating that the gums obtained from these byproducts can be successfully applied in an emulsion product, such as mayonnaise and salad dressing.

The oil particle size (d_32_) and PdI value of the samples were found as 1240.00–8581.33 μm, and 0.249–0.701, respectively. The researchers emphasized that the oil particle diameter decreased significantly as the polysaccharide concentration increased [78,79]. As can be seen, the mayonnaise samples containing AG and RG exhibited lower particle size and PdI value as compared to other mayonnaise samples. Also, all samples have a sufficient zeta potential value.

#### 3.5.3. Oxidative Stability (at 90 °C)

The oxidative stability of the vegan mayonnaise samples was determined with the Oxitest device, and the IP values of the samples were recorded. Table 8 showed the IP values of the samples. The IP values varied between 4:38 and 13:44 h. The sample prepared with xanthan gum showed the lowest IP value. The IP values of the samples prepared with gums obtained from byproducts were higher than the vegan mayonnaise samples prepared with xanthan gum. The IP value of the sample prepared with RSG was much higher than other samples. The very high IP value of the sample prepared with RSG can be explained by the RSG concentration. Due to the low consistency of RSG, it was used at the level of 5%. During the extraction of RSG, antioxidant products, such as phenolic compounds, may have transferred to gum solution and caused higher IP value. The higher IP value of FSG and RSG than xanthan gum. This result indicated that gums obtained from byproducts could exhibit a favorable condition in terms of oxidative stability as well as providing desirable consistency.

## 4. Conclusions

In recent years, cold-pressed oil consumption has increased due to the bioactive components in its structure. After the consumption of cold-pressed oil, a byproduct rich in protein, carbohydrates, and bioactive components emerges. Adding value to these byproducts is an important issue for the cold press oil industry. In this study, the production of gum from cold-pressed flax, chia, and rocket seed oil wastes and the use of the obtained gum in vegan mayonnaise were investigated. FSG and CSG are rich in xylose and glucose, while RSG is rich in galactose and mannose. While FGS and CSG exhibited high pseudoplastic, viscoelastic solid, and recoverable structure at low concentrations, RSG exhibited these properties at high concentrations. The potential for use as stabilizers and emulsifiers in the production of FSG, CSG, and RSG vegan mayonnaise has been investigated. The results of the study showed that FSG, CSG, and RSG can provide desired rheological and microstructural properties and oxidative stability in the production of reduced-fat vegan mayonnaise. The results of this study indicated that this study could offer a new perspective in adding value to flaxseed, chia seed, and rocket seed cold-press oil by-product.

## Figures and Tables

**Table 1:** Physicochemical properties of gum.

	CSG	FSG	RSG
Carbohydrate (%*w*/*w*)	73.59 ± 0.29 ^b^	78.56 ± 0.06 ^a^	70.48 ± 0.20 ^c^
Protein (%*w*/*w*)	9.45 ± 0.07 ^b^	11.38 ± 0.10 ^a^	11.00 ± 0.01 ^a^
Fat (%*w*/*w*)	1.01 ± 0.02 ^b^	2.16 ± 0.10 ^a^	1.94 ± 0.04 ^a^
Moisture (%*w*/*w*)	9.60 ± 0.18 ^s^	9.08 ± 0.06 ^b^	9.95 ± 0.08 ^a^
Ash (%*w*/*w*)	6.26 ± 0.14 ^b^	7.89 ± 0.11 ^a^	6.63 ± 0.05 ^b^
Monosaccharides (%)			
Glucose	20.46 ± 0.16 ^a^	20.27 ± 0.15 ^a^	10.26 ± 0.06 ^b^
Galactose	6.70 ± 0.07 ^c^	19.91 ± 0.03 ^b^	22.08 ± 0.08 ^a^
Mannose	4.09 ± 0.01 ^b^	0.41 ± 0.01 ^c^	39.12 ± 0.12 ^a^
Xylose	36.15 ± 0.15 ^b^	38.75 ± 0.05 ^a^	-

CSG: chia seed byproduct gum, FSG: flaxseed byproduct gum, RSG: rocket seed byproduct gum. The different lowercase letter in the same line indicates statistical significance (*p* < 0.05).

**Table 2:** Steady shear power-law parameters of gum solutions.

	Gum (%)	K (Pa·s^n^)	*n*	R^2^
FSG-1	1.0	8.256 ± 0.043 ^C^	0.143 ± 0.000	0.942
FSG-1.5	1.5	15.699 ± 0.120 ^B^	0.143 ± 0.001	0.972
FSG-2	2.0	28.731 ± 0.341 ^A^	0.145 ± 0.001	0.956
CSG-1	1.0	18.578 ± 0.087 ^C^	0.296 ± 0.011	0.998
CSG-1.5	1.5	27.889 ± 0.092 ^B^	0.243 ± 0.003	0.995
CSG-2	2.0	49.028 ± 0.0359 ^A^	0.252 ± 0.001	0.994
RSG-1	1.0	0.209 ± 0.001 ^E^	0.460 ± 0.004	0.999
RSG-1.5	1.5	0.694 ± 0.015 ^D^	0.342 ± 0.003	0.997
RSG-2	2.0	0.985 ± 0.002 ^C^	0.313 ± 0.001	0.996
RSG-3	3.0	1.217 ± 0.003 ^B^	0.305 ± 0.000	0.998
RSG-5	5.0	3.553 ± 0.021 ^A^	0.224 ± 0.001	0.987

CSG: chia seed byproduct gum, FSG: flaxseed byproduct gum, RSG: rocket seed byproduct gum. A different uppercase letter in the same column indicates statistical significance (*p* < 0.05).

**Table 3:** Weltman and second-order structural kinetic model parameters defining the time-dependent flow behavior of gum solutions at 25 °C.

		Weltman Model			Second-Order Structural Model				
	Gum (%)	A (Pa)	-B (Pa)	R^2^	k ∗ 1000	*η*_0_ (Pa·s)	*η_e_* (Pa·s)	*η*_0_/*η_e_*	R^2^
FSG-1	1.0	4.910 ^C^	0.133 ^C^	0.984	3.666 ^C^	23.346 ^C^	5.276 ^C^	4.425 ^C^	0.996
FSG-1.5	1.5	8.911 ^B^	0.193 ^B^	0.986	7.641 ^B^	59.080 ^B^	6.415 ^B^	9.209 ^B^	0.993
FSG-2	2.0	19.923 ^A^	0.683 ^A^	0.996	13.522 ^A^	138.530 ^A^	9.166 ^A^	15.113 ^A^	0.997
CSG-1	1.0	13.988 ^C^	0.550 ^C^	0.921	5.566 ^C^	74.176 ^C^	11.842 ^C^	6.264 ^C^	0.997
CSG-1.5	1.5	20.407 ^B^	0.661 ^B^	0.952	7.480 ^B^	256.477 ^B^	15.567 ^B^	16.475 ^B^	0.996
CSG-2	2.0	29.349 ^A^	0.701 ^A^	0.999	14.329 ^A^	423.919 ^A^	20.661 ^A^	20.517 ^A^	0.994
RSG-1	1.0	0.281 ^E^	0.012 ^E^	0.966	1.309 ^E^	1.219 ^D^	0.947 ^E^	1.287 ^E^	0.998
RSG-1.5	1.5	0.309 ^D^	0.017 ^D^	0.992	3.157 ^D^	6.626 ^C^	3.976 ^B^	1.668 ^D^	0.999
RSG-2	2.0	0.731 ^C^	0.041 ^C^	0.993	6.787 ^C^	8.837 ^B^	4.466 ^A^	1.978 ^C^	0.990
RSG-3	3.0	2.195 ^B^	0.137 ^B^	0.987	8.602 ^B^	8.953 ^B^	3.092 ^C^	2.896 ^B^	0.992
RSG-5	5.0	3.281 ^A^	0.212 ^A^	0.996	10.656 ^A^	17.499 ^A^	2.899 ^D^	6.036 ^A^	0.993

CSG: chia seed byproduct gum, FSG: flaxseed byproduct gum, RSG: rocket seed byproduct gum; constant shear rate (0.5 s^−1^). A different uppercase letter in the same column indicates statistical significance (*p* < 0.05).

**Table 4:** Second-order structural kinetic model parameters for 3-ITT.

	*G*_0_	*G_e_*	*G_e_*/*G*_0_	k × 1000	R^2^	% D	% R
FSG-1	1.285 ± 0.011 ^C^	1.542 ± 0.107 ^C^	1.200 ^C^	7.41 ^C^	0.970	53.79	55.47
FSG-1.5	2.694 ± 0.049 ^B^	3.390 ± 0.012 ^B^	1.259 ^B^	35.56 ^B^	0.998	61.35	48.64
FSG-2	6.368 ± 0.124 ^A^	8.200 ± 0.300 ^A^	1.288 ^A^	56.71 ^A^	0.997	53.52	59.85
CSG-1	38.788 ± 1.561 ^C^	59.525 ± 0.565 ^C^	1.535 ^C^	33.27 ^C^	0.909	16.41	128.29
CSG-1.5	44.234 ± 0.095 ^B^	75.015 ± 0.352 ^B^	1.696 ^B^	38.99 ^B^	0.990	38.05	61.95
CSG-2	76.001 ± 0.219 ^A^	133.000 ± 0.435 ^A^	1.750 ^A^	41.80 ^A^	0.948	36.13	111.76
RSG-1	0.461 ± 0.003 ^E^	0.518 ± 0.019 ^E^	1.124 ^E^	11.00 ^E^	0.983	17.67	256.47
RSG-1.5	0.744 ± 0.014 ^D^	0.842 ± 0.005 ^D^	1.132 ^D^	14.91 ^D^	0.980	46.47	89.33
RSG-2	0.800 ± 0.020 ^C^	0.914 ± 0.259 ^C^	1.143 ^C^	7.08 ^C^	0.983	50.62	99.60
RSG-3	1.268 ± 0.209 ^B^	1.557 ± 0.083 ^B^	1.228 ^B^	8.56 ^B^	0.996	47.89	105.48
RSG-5	4.617 ± 0.308 ^A^	5.869 ± 0.438 ^A^	1.271 ^A^	9.47 ^A^	0.998	65.59	81.91

CSG: chia seed byproduct gum, FSG: flaxseed byproduct gum, RSG: rocket seed byproduct gum. % D: % Deformation and % R: %Recovery. A different uppercase letter in the same column indicates statistical significance.

**Table 5:** Power-law parameters of dynamic rheological properties of the CSG, FSG, and RSG solutions.

	K′ (Pa·s)	n′	R^2^	K″ (Pa·s)	n″	R^2^
FSG-1	0.949 ± 0.002 ^C^	0.358 ± 0.002	0.9994	0.217 ± 0.004 ^C^	0.656 ± 0.069	0.9981
FSG-1.5	2.933 ± 0.025 ^B^	0.285 ± 0.015	0.9975	1.049 ± 0.006 ^B^	0.459 ± 0.010	0.9880
FSG-2	5.666 ± 0.031 ^A^	0.279 ± 0.005	0.9997	2.216 ± 0.033 ^A^	0.453 ± 0.006	0.9985
CSG-1	10.679 ± 0.031 ^C^	0.326 ± 0.002	0.9978	7.328 ± 0.012 ^C^	0.326 ± 0.002	0.9917
CSG-1.5	14.655 ± 0.093 ^B^	0.304 ± 0.001	0.9990	11.013 ± 0.307 ^B^	0.304 ± 0.011	0.9964
CSG-2	24.849 ± 0.101 ^A^	0.212 ± 0.000	0.9992	19.543 ± 0.076 ^A^	0.287 ± 0.000	0.9807
RSG-1	0.011 ± 0.001 ^E^	0.118 ± 0.103	0.9993	0.007 ± 0.000 ^E^	0.117 ± 0.079	0.9989
RSG-1.5	0.074 ± 0.002 ^D^	0.809 ± 0.098	0.989	0.060 ± 0.003 ^D^	0.801 ± 0.106	0.988
RSG-2	0.151 ± 0.002 ^C^	0.679 ± 0.045	0.9987	0.150 ± 0.034 ^C^	0.721 ± 0.009	0.9983
RSG-3	0.232 ± 0.001 ^B^	0.508 ± 0.002	0.9899	0.219 ± 0.019 ^B^	0.720 ± 0.032	0.9983
RSG-5	2.178 ± 0.041 ^A^	0.202 ± 0.000	0.9982	0.634 ± 0.092 ^A^	0.213 ± 0.001	0.9910

CSG: chia seed byproduct gum (1–2%), FSG: flaxseed byproduct gum (1–2%), RSG: rocket seed byproduct gum (1–5%); K′ and K″: consistency coefficient (Pa·s^n^); n′ and n″: flow behavior index values; R^2^: determination of coefficient. A different uppercase letter in the same column indicates statistical significance.

**Table 6:** Rheological properties of low-fat vegan mayonnaise samples * with a different type of gum.

	Gum (%)	K (Pa·s^n^)	R^2^		
CG-VM	2.0	38.582 ± 0.183 ^A^	0.985		
FG-VM	2.0	35.913 ± 0.238 ^B^	0.986		
RG-VM	5.0	10.647 ± 0.232 ^C^	0.999		
AG-VM	1.0	0.007 ± 0.002 ^E^	0.997		
GG-VM	1.0	10.888 ± 0.313 ^C^	0.994		
XG-VM	0.4	7.050 ± 0.069 ^D^	0.994		
	K′ (Pa·s^n^)	n′	K″ (Pa·s^n^)	n″	R^2^
CG-VM	43.317 ± 0.098 ^A^	0.299 ± 0.000	30.423 ± 0.032 ^A^	0.094 ± 0.005	0.9973
FG-VM	31.321 ± 0.291 ^C^	0.309 ± 0.001	11.688 ± 0.020 ^C^	0.370 ± 0.016	0.9960
RG-VM	32.511 ± 0.310 ^C^	0.191 ± 0.009	5.534 ± 0.197 ^D^	0.301 ± 0.002	0.9835
AG-VM	0.012 ± 0.001 ^E^	0.992 ± 0.031	0.006 ± 0.000 ^F^	1.201 ± 0.021	0.9993
GG-VM	36.752 ± 0.180 ^B^	0.264 ± 0.002	12.489 ± 0.129 ^B^	0.361 ± 0.024	0.9790
XG-VM	12.721 ± 0.201 ^D^	0.274 ± 0.008	4.364 ± 0.082 ^E^	0.238 ± 0.007	0.9941
	*G*_0_	*G_e_*	*G_e_*/*G*_0_	k × 1000	R^2^
CG-VM	64.613	104.270	1.614 ^A^	60.789 ^B^	0.9844
FG-VM	53.781	70.591	1.313 ^D^	37.57 ^C^	0.9990
RG-VM	38.071	47.951	1.260 ^E^	24.987 ^E^	0.9874
AG-VM	0.258	0.180	0.698 ^F^	4.32 ^F^	0.9938
GG-VM	65.192	97.138	1.490 ^B^	68.876 ^A^	0.9974
XG-VM	14.478	20.176	1.394 ^C^	29.90 ^D^	0.9981

CG-VM: vegan mayonnaise contained chia seed byproduct gum, FG-VM: vegan mayonnaise contained flaxseed byproduct gum, RG-VM: vegan mayonnaise contained rocket seed byproduct gum, AG-VM: vegan mayonnaise contained Arabic gum, GG-VM: vegan mayonnaise contained guar gum, XG-VM: vegan mayonnaise contained xanthan gum; K, K′, and K″: consistency coefficient (Pa·s^n^); n, n′, and n″: flow behavior index values; *G*_0_: the initial values of the storage modulus; *G_e_*: the equilibrium storage modulus; k: the rate constant of recovery of the sample; R^2^: determination of coefficient. * low-fat vegan mayonnaise samples contain 30% vegetable oil and 1% lecithin. A different uppercase letter in the same column indicates statistical significance.

**Table 7:** The zeta potential and particle size of vegan mayonnaise samples with a different type of gum.

	ζ-Potential (mV)	PdI	d_32_ (µm)
CG-VM	−42.0 ± 1.58 ^A^	0.507 ± 0.121 ^D^	5418.00 ± 268.88 ^C^
FG-VM	−40.4 ± 1.79 ^A^	0.701 ± 0.050 ^A^	8581.33 ± 284.39 ^A^
RG-VM	−39.5 ± 1.21 ^A^	0.302 ± 0.000 ^E^	3689.67 ± 282.13 ^D^
AG-VM	−31.9 ± 0.62 ^B^	0.249 ± 0.048 ^F^	1240.00 ± 102.77 ^E^
GG-VM	−42.8 ± 1.04 ^A^	0.539 ± 0.092 ^C^	5851.33 ± 114.01 ^C^
XG-VM	−41.2 ± 0.89 ^A^	0.613 ± 0.074 ^B^	6720.32 ± 160.99 ^B^

CG-VM: vegan mayonnaise contained chia seed byproduct gum, FG-VM: vegan mayonnaise contained flaxseed byproduct gum, RG-VM: vegan mayonnaise contained rocket seed byproduct gum, AG-VM: vegan mayonnaise contained Arabic gum, GG-VM: vegan mayonnaise contained guar gum, XG-VM: vegan mayonnaise contained xanthan gum. ζ-Potential: zeta-potential (mV); PdI: polydispersity index; d_32_: the oil particle size (µm). A different uppercase letter in the same column indicates statistical significance.

**Table 8:** The induction period (IP (h)) of vegan mayonnaise samples with a different type of gum at 90 °C.

Samples	IP (h)
CG-VM	6:15 ± 0.02 ^B^
FG-VM	5:35 ± 0.08 ^D^
RG-VM	13:44 ± 0.12 ^A^
AG-VM	6:00 ± 0.01 ^C^
GG-VM	5:27 ± 0.04 ^D^
XG-VM	4:38 ± 0.03 ^E^

CG-VM: vegan mayonnaise contained chia seed byproduct gum, FG-VM: vegan mayonnaise contained flaxseed byproduct gum, RG-VM: vegan mayonnaise contained rocket seed byproduct gum, AG-VM: vegan mayonnaise contained Arabic gum, GG-VM: vegan mayonnaise contained guar gum, XG-VM: vegan mayonnaise contained xanthan gum. IP: induction period (h). A different uppercase letter in the same column indicates statistical significance.

## Data Availability

The data presented in this study are available on request from the corresponding author.
